# Induced Coma, Death, and Organ Transplantation: A Physiologic, Genetic, and Theological Perspective

**DOI:** 10.3390/ijms24065744

**Published:** 2023-03-17

**Authors:** Cezar-Ivan Coliță, Denissa-Greta Olaru, Daniela Coliță, Dirk M. Hermann, Eugen Coliță, Daniela Glavan, Aurel Popa-Wagner

**Affiliations:** 1 Doctoral School, University of Medicine and Pharmacy Carol Davila, 020276 Bucharest, Romania; cezar.ivan23@gmail.com (C.-I.C.);; 2 Department of Psychiatry, University for Medicine and Pharmacy Craiova, 200349 Craiova, Romania; denissagretaolaru@gmail.com; 3 Chair of Vascular Neurology, Dementia and Ageing, Department of Neurology, University Hospital Essen, 45147 Essen, Germany

**Keywords:** death, genetics, cellular proliferation, apoptosis, proteasomal degradation, cancer, organ transplantation, theology

## Abstract

In the clinic, the death certificate is issued if brain electrical activity is no longer detectable. However, recent research has shown that in model organisms and humans, gene activity continues for at least 96 h postmortem. The discovery that many genes are still working up to 48 h after death questions our definition of death and has implications for organ transplants and forensics. If genes can be active up to 48 h after death, is the person technically still alive at that point? We discovered a very interesting parallel between genes that were upregulated in the brain after death and genes upregulated in the brains that were subjected to medically-induced coma, including transcripts involved in neurotransmission, proteasomal degradation, apoptosis, inflammation, and most interestingly, cancer. Since these genes are involved in cellular proliferation, their activation after death could represent the cellular reaction to escape mortality and raises the question of organ viability and genetics used for transplantation after death. One factor limiting the organ availability for transplantation is religious belief. However, more recently, organ donation for the benefit of humans in need has been seen as “posthumous giving of organs and tissues can be a manifestation of love spreading also to the other side of death”.

## 1. Introduction

The word “death” is derived from the Greek word “thanatos” [θάνατος] and refers to both a physical punishment or spiritual punishment [Rom. 6:23]. Clinical death may have different meanings. Classically, cessation of vital functions, including cessation of heartbeat and respiration are the two criteria that are necessary to declare a person dead. In the clinic, the death certificate is issued if brain electrical activity is no longer detectable.

*The concept of brain death has been challenged by the Church. However, in 2015 the German Bishops Conference stated that brain death is the best and safest criterion to declare a person dead*.

After death, some of the organs may still be alive and can be used for transplantation. Quality criteria for the organs that are used for transplantation include organ pathophysiology and metabolic viability. Although gene expression is more sensitive than pathophysiological changes, the organs that are used for transplantation are not tested for gene expression or epigenetic modifications. However, gene activity does not cease after a person is declared dead, questioning the very definition of death. Indeed, the discovery that many genes are still working up to 48 h after death [1,2,3,4] has implications for our very definition of death, and in particular for forensics and organ transplantation.

## 2. Ex Vivo Organ Viability and Gene Expression

### 2.1. The Heart

Heart viability after death is short, 4–6 h. Currently, in the clinic, only hearts from donors meeting brain death criteria are accepted for heart transplantation. Indeed, modern resuscitation methods and availability of modern mechanical life-supporting systems have required the introduction of the concept of brain death. Thus, death by neurologic criteria means permanent cessation of the brainstem and cerebral functions, including no respiratory drive and lack of response to stimulation of the cranial nerve or to pain stimuli. For an adult patient who received a heart transplant, the median survival time following heart transplantation now approaches 12.2 years. More recently, because of increased demand, heart organs that are obtained from donors following circulatory death, i.e., the irreversible cessation of all circulatory and respiratory function, are also being used [5,6,7,8]. 

Gene set enrichment analysis and gene ontology analysis were used to study the transcriptional profile of the human left ventricle (LV) and right ventricle (RV) after 0, 4, and 8 h of cold ischemic storage in a preservation solution. Quite surprisingly, the LV and RV showed distinct, opposing genetic responses to cold storage including changes related to inflammation, including NFκB activation, oxidative phosphorylation, and fatty acid metabolism pathways after 8 h of storage [9]. 

### 2.2. The Lung

Lung viability after death is similar to that of the heart, i.e., 4–6 h. Unfortunately, brain death leads to hemodynamic neuroendocrine abnormalities and metabolic decline, causing neurogenic pulmonary edema that limits the viability of the lungs. Since the percentage of lung retrieval rate is lower when compared to other organs that are used for transplantation, lungs after cardiocirculatory death (DCD) and brain death are increasingly used in the clinic [10,11,12]. Gene expression in ex vivo normothermic perfused lungs has revealed increases in endothelial markers of inflammation and decreases in circulating leukocyte transcripts coding for CCL2, CCL3, CCR1, and CCR2, suggesting that lung recovery after normothermic perfusion follows specific stages, i.e., cellular death, cellular preservation, cellular reorganization, and cellular invasion [13].

### 2.3. The Liver

The liver can survive a longer time outside of the living body (8–12 h) and both hypothermic and normothermic perfusion machines are being used to extend the liver viability up to 24 h. Normothermic machine perfusion (NMP) used for liver preservation provides a near-physiological environment to the liver and maintains the liver at 37 °C in a physiological state through the delivery of oxygen and nutrients. NMP allows organ therapy and quantitation of many metabolic and dynamic parameters to assess graft quality and viability. Hypothermic machine perfusion used for preservation in a cold environment relies on the reduction of the metabolic rate and, therefore, does not allow assessment of graft viability that is usually done by measuring lactate production, biliary biomarker, or transaminases [14].

A common stressor to all organs after stress is the lack of oxygen. Thus, global gene expression profiles in liver biopsies after cold storage and at 90 min postreperfusion using the Affymetrix GeneChip Human Gene 1.0 ST array revealed significant alteration in transcripts coding for antioxidant, immunological, lipid biosynthesis, cell development, and growth pathways [15].

### 2.4. The Kidney

Kidney viability after death is considerably long, 24–36 h. Similarly, both hypothermic and normothermic perfusion machines are being used to preserve and extend kidney viability. However, the major limiting factor for using kidneys after death is circulatory death causing kidney ischemia, organ damage, and viability impairment. One approach to preserve kidney viability is to keep the kidney, after being washed of blood, in a cold solution and attached to a hypothermic working machine at 4 °C. At this temperature, the rate of metabolism is 10% of that at normal physiological temperature. Gene expression analysis of biopsies that were taken from kidneys of deceased persons and kept in the cold solution has shown that indeed inflammation that was caused by cold ischemia was the major factor for transplant rejection at 3 months. Other activated genetic pathways were related to cell cycle/growth (e.g., IGFBP5, CSNK2A2), signal transduction (e.g., RASGRP3), immune response (e.g., CD83, BCL3, MX1), and metabolism (e.g., ENPP4, GBA3) [16,17].

### 2.5. The Brain

Brains cannot be transplanted. Therefore, information about postmortem transcriptomics was obtained in a different way. Thus, to mimic the postmortem interval, the transcription patterns and histological features of postmortem brains were compared to slices of healthy cortical tissue surrounding the epileptic focus that were obtained immediately at surgery of epileptic patients. Within a few hours, a selective reduction in the transcriptional activity of neuronal genes with relative preservation of housekeeping gene expression was recorded, suggesting that cessation of brain electrical activity is a valid criterion for brain death. However, at the same time, there was a reciprocal increase in astroglial and microglial gene expression, resulting in the conversion to a reactive phenotype that increased gradually for at least 24 h after tissue removal. Histologically, neurons were rapidly degenerating and caused strong glial cell reactivity by activating their processes [4]. 

### 2.6. Death Genes in Animal Models

More detailed technical studies of the death genes are available in animal models such as mice and zebrafish. A complex genetic analysis has shown that in postmortem tissues from animal models, 99% of gene transcripts decreased in abundance within 30 min after death while 1% had increased in abundance for up to 96 h postmortem. Quite interestingly, by function, the most abundant transcripts were involved in transport, stress, apoptosis, immunity, inflammation, development, epigenetic regulation, and cancer [1]. Remarkably, this pattern of gene expression in animal models was tissue-specific. Thus, in the mouse, brain transcripts increased in the first hour and then gradually decreased, while mRNA gradually decreased with increased postmortem time (PMT) in the mouse liver suggesting that mRNAs are more stable in the brain than in the liver [18,19]. Similar findings have been reported for humans, i.e., genes that are involved in wound healing and contracting heart muscle were active for at least 12 h after death in people who had died from multiple trauma, heart attack, or suffocation [20].

### 2.7. Gene Expression in Deep Coma

We asked if there are similarities between gene expression in the brain after death and gene expression in induced coma. By searching the available literature, we found that indeed, seven genes were expressed both in the postmortem brains and induced coma: *Cdc42*, *Csnk2a1*, *Gadd45a*, *Cdc42*, *Tnfrsf14*, *Prdx2*, *Tpr*, *Rasa1*, and four genes had a similar function both in the postmortem brains and induced coma, *Klkb1*, *Bcl2*, *Ier3*, and *Zfand2a*. By functionality, we grouped genes according to their function: regulator of energy metabolism (*Gadd45a*), apoptosis (*Bcl2*, *Ier3*), catabolic processes (*Zfand2a*), inflammation (*Klkb1*, *Tnfrsf14*), cell cycle control, and cancer (*Csnk2a1*, *Cdc42*, *Rasa1*, *Prdx2*, *Tpr*) (Table 1).

*Gadd45a* encodes the growth arrest and DNA damage-inducible protein 45 alpha, a key regulator of energy metabolism and longevity by regulating epigenetic DNA methylation [21]. *Gadd45a* transcript levels are also increased under stressful conditions [22]. *Gadd45* family members regulate this function through multiple cellular processes (e.g., DNA demethylation, gene expression, RNA stability, MAPK signaling) [23]. During development, the *Gadd45a* gene is required for neural cell proliferation and is used as a pan-neural and neural crest marker [24,25]. It has been hypothesized that GADD45 proteins are essential for brain function and their dysfunction might underlie pathophysiological conditions such as neuropsychiatric disorders and misexpression of Gadd45 family members were associated with psychiatric diseases [23]. Thus, unpredictable chronic mild stress (UCMS) reduces the expression of Gadd45 family members, suggesting that Gadd45 family members are new putative targets for UCMS treatment [26].

*Bcl2* gene encodes an integral outer mitochondrial membrane protein that blocks the apoptotic death of some cells, including lymphocytes. Constitutive expression of BCL2 is thought to be the cause of follicular lymphoma, mature T-cell lymphomas [27], prostate cancer [28], and renal cell carcinomas [29]. Most importantly for the brain death, stress activates cell survival and cell death signaling pathways that converge on mitochondria, a process that is controlled by the activities of BCL-2 proteins. Indeed, BCL-2 proteins play a significant role in initiating or inhibiting apoptosis during neuronal development and injury. Emerging evidence suggests that BCL-2 is involved in maintaining both mitochondrial bioenergetics and neuronal membrane potential by modulation of Ca^2+^ signaling during acute neuronal injury and thus plays a role in neuroprotection [30].

*Ier3* is a stress inducible gene that encodes for an immediate early response 3 (IER3) protein that is involved in the protection of cells from *Fas*- or tumor necrosis factor type alpha-induced apoptosis. Increasing evidence suggests that IER3 functions either as an oncogene or as a tumor suppressor in various human cancers [31]. Thus, it was recently reported that IER3 induces the apoptosis of cervical cancer cells and that its expression is downregulated in patients with cervical cancer via its ubiquitination, followed by proteasomal degradation [32,33].

The *Zfand2a* mRNA is normally strongly expressed in the eye, brain, and heart of mice. The ZFAND2A/AIRAP protein encoded by Zfand2a mRNA is part of the proteasome complex and is involved in proteasome-mediated ubiquitin-dependent accelerated protein catabolic processes in the skeletal muscles and protein targeting to ER, resulting in a loss of muscle mass by increasing ATP hydrolysis [34,35,36]. Therefore, pharmacological antagonists of ZFAND5 may serve as a treatment of the debilitating loss of muscle protein in a variety of cachectic disorders, as well as in these muscular dystrophies [37]. A recent study also identified ZFAND2A/AIRAP as a novel stress-regulated survival factor implicated in the stabilization of the antiapoptotic protein cIAP2 that is highly expressed in several cancers, including melanoma. An increased expression of cIAP2 blocks the pro-apoptotic pathways and increases resistance to the drug bortezomib in melanoma cells. Since ZFAND2A/AIRAP-siRNA-mediated knockdown affects cIAP2 protein stability in melanoma cells, ZFAND2A/AIRAP downregulation markedly enhances bortezomib anticancer activity in melanoma [38].

*Klkb1* transcript encodes a glycoprotein that is part of the plasma contact activation system and plays a role in the defense response to infections by participating in the surface-dependent activation of blood coagulation, fibrinolysis, and kinin generation. Indeed, it was recently shown that kallikrein (KK) activates the complement system by cleaving C3 and factor B, with subsequent formation of an active complement C3 convertase to yield active components C3a and C3b and trigger complement activation [39]. Recent evidence suggests that kallikrein-kinin and the coagulation system contribute to autoimmune CNS diseases such as multiple sclerosis (MS) by mediating transendothelial trafficking of inflammatory cells across the blood brain barrier (BBB). Since pharmacological inhibition of plasma prekallikrein (PKK) or KK, respectively, protects mice from experimental autoimmune encephalomyelitis (EAE) by reducing transendothelial leukocyte trafficking, PK inhibition may offer a strategy for the treatment of MS [40].

*Tnfrsf14* transcript encodes a member of the tumor necrosis factor (TNF) receptor superfamily that has a pivotal role in T-cell-mediated adaptive immunity and immune diseases. The encoded protein functions in signal transduction pathways that activate inflammatory and inhibitory T-cell immune responses [41]. TNFR superfamily molecules including TNFRSF14, are constitutive or inducible expressed on T cells and play important roles in protective immunity, inflammatory, autoimmune diseases, and tumor immunotherapy [42]. In the brain, *Tnfrsf14* transcripts showed a positive correlation with a WHO grade of glioma and TNFRSF14 was significantly increased in a mesenchymal glioma subtype. Indeed, a higher TNFRSF14 expression was significantly associated with a shorter survival for glioma patients and Cox regression models revealed that TNFRSF14 expression was an independent variable for predicting survival [43]. 

One gene that is shared by both conditions is *Csnk2a1*. *Csnk2a* mRNA encodes casein kinase II, a constitutively active serine/threonine protein kinase that phosphorylates hundreds of acidic proteins, including casein. It is involved in various signaling pathways and cellular processes, including cell cycle control, apoptosis, and circadian rhythmicity. In the brain, casein kinase 2 (CK2), is highly expressed in infections, Alzheimer’ disease (AD) patients, and contributes to the AD pathology by regulating NR2B-mediated neurotransmission [44]. CK2 has enhanced expression/activity in a plethora of human diseases and numerous cancers, including glioblastoma (GBM). Therefore, inhibitors of CK2 may be good candidates for the inhibition of tumor growth as has been shown in GBM xenograft mouse models [45,46].

*Cdc42* transcript encodes a small GTPase of the Rho-subfamily, which regulates signaling pathways that control diverse cellular functions including cell morphology, migration, endocytosis, cell cycle progression, and epigenetic regulation. Indeed, CDC42 can downregulate the inhibitor of DNA binding 4 (ID4) which has been shown to be overexpressed in colorectal adenocarcinomas through hypermethylation of its promoter [47]. In the brain, the small GTPase, CDC42, plays an essential role in neurogenesis and brain development. Thus, CDC42 stimulates mTORC1 activity and thereby upregulates tissue-specific transcription factors that are essential for neural progenitor formation. Further, by promoting EGFR degradation, it was found that CDC42b and ACK stimulate autophagy and trigger neural progenitor cells to differentiate into neurons [48].

The protein that is encoded by *Rasa1* mRNA is part of the GAP1 family of GTPase-activating proteins which regulate multiple cellular signaling pathways including those that control cell growth, differentiation, and survival. RASA1 is involved in numerous physiological processes such as angiogenesis, cell proliferation, and apoptosis. Mutations leading to changes in the binding sites of RASA1 are associated with basal cell carcinomas [49] and multiple tumor types of the lung, intestines, liver, and breast [50].

*Prdx2* gene encodes a member of the peroxiredoxin family of antioxidant enzymes, that reduce hydrogen peroxide and alkyl hydroperoxides and thus plays an antioxidant protective role in cells. Thus, PRDX2 reduces the production of reactive oxygen species by catalyzing the reduction of hydrogen peroxide to water, thereby protecting neurons against oxidative stress. However, overexpression of PRDX2 accelerates brain damage after stroke by activating an inflammatory response [51]. Therefore, restoration of redox balance may play an important role in minimizing the detrimental effects of oxidative damage in neurodegenerative disorders. PRDX2 is also an antioxidant and molecular chaperone that can be secreted by tumor cells. Increasing evidence suggests that the role of PRDXs may go beyond the antioxidant properties and could be related to the regulation of cell signaling. Thus, induction of oxidative stress via H_2_O_2_ treatment leads to the overexpression of PRXD-2 and inhibition of cancer cell proliferation [52,53]. Recent results also indicate that PRDX2 promotes cell survival and inflammation-associated myocardial hypertrophy [54].

*Tpr* gene encodes a large coiled-coil protein that forms intranuclear filaments that are attached to the inner surface of nuclear pore complexes (NPCs). During oncogene-induced senescence (OIS), heterochromatin forms internal senescence-associated heterochromatin foci (SAHFs). Recently it was shown that the nucleoporin TPR is necessary for both the formation and maintenance of SAHFs [55]. Of note, TPR expression is significantly increased in lung cancer tissues and correlated with poor prognosis [56]. 

## 3. Cell Viability and Metabolic Responses in Induced Coma and Postmortem Tissue

Clinical trials have shown that in human patients, prolonged induced coma in intensive care units is associated with cognitive impairment in survivors [56,57]. Yet, at the synapse-level, a morphological correlation between prolonged anesthesia and cognition could be found only in mice that were subjected to prolonged general anesthesia. Thus, the authors used two-photon imaging of fluorescently labeled dendrites and synapses to assess synaptic turnover and found that synapse turnover was more than doubled in experimental anesthesia. An increased synapse turnover may result in impaired cognition [58].

There is a common belief that most organelles, including mitochondria, deteriorate after death. Neuronal function in the brain requires a lot of energy and a reduction in ATP production in neurons can negatively affect neuronal function. Indeed, numerous studies have reported altered or decreased mitochondrial energy production in neurodegenerative diseases, neurometabolic disorders such as Leigh syndrome, and in psychiatric disorders such as schizophrenia and bipolar disorder [59,60,61,62,63,64]. However, it has been reported that there are plenty of structurally intact and functional mitochondria in postmortem mouse and human brains shortly after death. Moreover, mitochondria can be frozen for future functional assessments [65].

Unfortunately, histological data that are obtained in studies on animals are often extrapolated to humans without considering the postmortem time. Autopsy tissue is rarely obtained within 2 h postmortem time (PMT). For example, in the mouse brain that was fixed at different PMT, significant metabolomic changes already occurred at 2 h PMT, whereas neuroanatomical changes occurred mostly at 5 h PMT. Thus, the levels of pyroglutamic acid, anandamide, and urea increased until 2 h PMT whereas GABA, creatinine, N-acetyl-aspartic acid, putrescine, and cadaverine reached higher levels at 5 h PMT. Other compounds such as cholesterol, cholesterol esters, and arachidonoylglycerol were stable during the study period [66]. Another study investigated the time course of postmortem proteolytic degradation of selected tissue antigens and found that the preservation of antigens for Ki67, Vimentin, Pancytokeratin, and CD20 was tissue-dependent, i.e., the autolysis process was not appreciable in the first 5 days PMT. However, the liver and the spleen underwent rapid autolysis, while the kidney displayed an autolysis of the tubules after one day PMT and parcellular autolysis of the glomeruli at 5 days PMT [67].

## 4. Discussion

In the clinic, the death certificate is issued if brain electrical activity is no longer detectable. The concept of brain death has been challenged by the Church. However, in 2015 the German Bishops Conference stated it is, nevertheless, the best and safest criterion to declare a person dead. In 1989 the Pontifical Academy of Science of the Roman Catholic Church also stated: “a person is dead when there has been total and irreversible loss of all capacity for integrating and coordinating physical and mental functions of the body as a unit” (https://sites.sju.edu/icb/position-catholic-church-organ-donation/, accessed on 14 March 2023).

The reliability of the encephalogram (EEG) to confirm brain death remains, nevertheless, controversial. Indeed, several studies have shown that brain electrical activity persists for up to 72 h after the person is declared dead [68,69]. 

**Figure 1:**
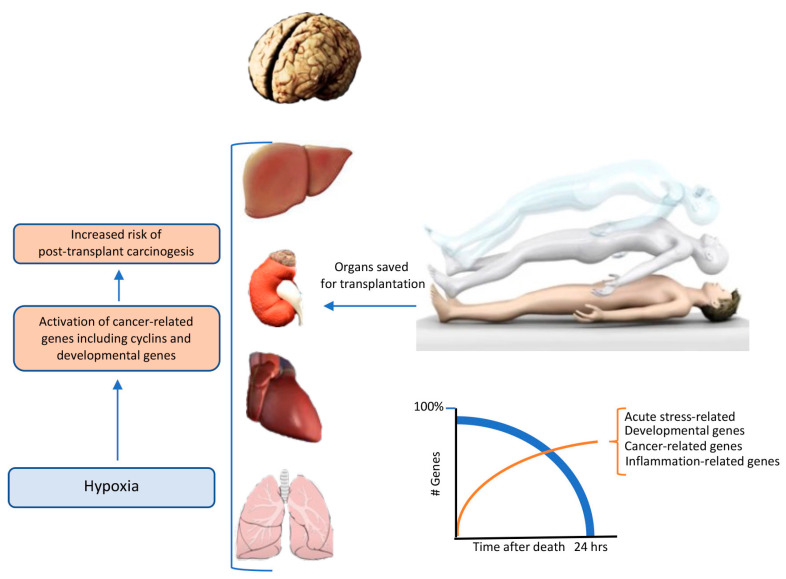
Time course of genetic events after cerebral death and their significance for solid organ transplantation. Within a few hours, there is a massive reduction in the transcriptional activity of neuronal genes with relative preservation of housekeeping genes that are commonly used as a reference for RNA normalization. However, about 1% of all transcripts are upregulated. By function, the most abundant transcripts were involved in transport, stress, apoptosis, immunity, inflammation, development, epigenetic regulation, and cancer.

By function, the most abundant transcripts were involved in transport, stress, apoptosis, immunity, inflammation, development, epigenetic regulation, and cancer [1]. Within a few hours, there is a selective reduction in the transcriptional activity of neuronal genes with relative preservation of housekeeping genes, commonly used as a reference for RNA normalization, suggesting that indeed, cessation of brain electrical activity could be taken as a valid death criterium (Figure 1).

So, why do so many genes become activated after death? One possible explanation is that these genes are involved in cellular proliferation during development. In every living body there is continuous competition for resources, even if we are not aware of it. Thus, a rapid decay of genes whose expression normally suppress other genes, such as those involved in embryological development, might allow the usually quiet genes to become active for a short period of time. Since these genes are involved in cellular proliferation, it could be an attempt of cells to escape mortality and raises the question of organ genetics and viability used for transplantation after death.

Secondly, once the inhibitory gene network disintegrates, it is natural for a cell to try escape mortality by proliferation. This immediately raises the next question about their long-term effects in the host. Since many such genes are shared both by the human brains after death and the brains in induced coma, it is legitimate to ask if long-term deep coma may have severe side effects that may result in cancer development. Another explanation is that many of the genes become active as part of physiological processes that aid healing or recovery after severe injury. For example, histological degeneration of neuronal structure during aging or brain injuries, is paralleled by activation of astroglia and microglia, a phenomenon that is seen both in the aging brain [70], Alzheimer disease [71], and brain injuries that are caused by stroke [72,73] or TBI [74].

Similarly, there were increases in abundance after death of genes that are required for innate (*Laao*, *Tox2*) and adaptive immunity (*Ms4a17*, *Usp18*) and increased levels of the apoptotic genes (*Fosb*, *Bcl2l11*) that are normally activated by infection and injury [75].

Third, death can be seen as a condition of extreme stress [2]. Hence, the increased abundance of specific transcripts shortly after death could be attributed to genes encoding proteins involved in resilient pathways (e.g., heat shock, *Hsp*, hypoxia-related, *Hif1ab* and oxidative stress, *Gadd45a*, *March4*) to compensate for extreme stress and increase survival chances [2].

A closely related phenomenon is the near-death experience [NDE] which is an altered state of consciousness due to the decoupling of the psychosoma from the physical body both in clinical conditions such as multiple organ failure, late-stage cancer confusion and life-threatening events including traffic accidents, traumatic brain injury (TBI), physical assaults, and drug abuse. NDEs are not a rare phenomenon, occurring in approximately 10–23% of cardiac arrest survivors and 3% of TBI survivors [76,77,78,79]. Since NDE has been known worldwide for a long time and numerous cultural backgrounds, it suggests an underlying common neurobiological mechanism. Thus, it has been hypothesized that thanatosis, aka death-feigning, or tonic immobility, is a highly conserved defense and survival mechanism that could have an evolutionary origin [80]. For example, when attacked by a predator, animals can feign death as a last resort to improve their chances of survival [81]. Indeed, in the animal kingdom, thanatosis is an anti-predator strategy that is part of an innate defense cascade [82,83] which is activated when mechanisms underlying the fight or flight response triggered by cortisol, are no longer possible [84,85,86,87,88]. Behaviorally, it involves sudden onset of immobility, with or without loss of tonic muscular activity, and unresponsiveness to external stimuli while awareness is preserved [84]. In humans, peritraumatic tonic immobility has been described as a cerebral defense mechanism in post-traumatic stress disorder that can happen during sexual assault [87,88,89].

Proposed mechanisms underlying NDEs include cortical spreading depolarizations (CSDs), migraine aura, a predictor of near-death experiences [90]. Indeed, a short-lasting variant of CSDs is considered the pathophysiological correlate of migraine aura [91] while terminal CSDs occur during the dying process of the brain in humans [92,93], rats [94], and insects [95]. At the molecular level, mechanisms underlying NDEs include the activity of the potent serotonergic psychedelic drug N,N-Dimethyltryptamine (DMT) [96] or hypofunction of the N-methyl-D-aspartate receptor (NMDAR) [96,97,98]. Quite interestingly, MDAR hypofunction is also induced by ketamine when used for recreational purposes [96], or serotonergic psychedelics (also referred to as serotonergic hallucinogens, psilocybin, LSD, and mescaline), including the endogenous serotonin 2A receptor agonist DMT, suggesting that endogenous NMDA antagonists with neuroprotective properties may be released in the proximity of death [99]. In a study that was published in the journal Nature, it was shown that in mice, ketamine produced 1–3 Hz oscillations in neurons of layer 5 of the retrosplenial cortex, a region that is required for visuospatial navigation and episodic memory, that blocked the communication with other brain parts. In humans, a 3 Hz oscillation in the deep posteromedial cortex (analog to mouse retrosplenial cortex) can induce dissociation in patients who experienced a seizure aura or have been stimulated externally [100].

We discovered a very interesting parallel between genes that were upregulated in the brain after death and genes that were upregulated in the brains subjected to induced coma, including genes involved in neurotransmission, proteosomal degradation, apoptosis, inflammation, and most interestingly, cancer. While in humans it is not possible to study gene activity related to these processes, it opens a new avenue of research on the side effects of induced coma in animal models.

Organs that are used for transplantation are mostly obtained from donors that are declared dead after irreversible loss of brain functions [101,102]. However, brain death causes a massive inflammatory response that triggers substantial circulatory and metabolic changes (e.g., hypoxia, pH) in the donor’s body that compromise organs vitality [103,104]. Indeed, biochemical investigations of biopsies that were obtained from organs used for transplantation have revealed substantial increases in the expression of genes that are involved in oxidative stress, apoptosis, adhesion, and inflammation biomarkers. Therefore, postmortem time could have a significant effect on gene expression because of the physiological changes that take place in the body after organismal death [105]. Yet to date, such studies have not been officially used to assess organ quality and/or transplantation success. Therefore, Pozhitkov and Noble [2] have suggested a closer examination of the relationship between gene expression and postmortem time which might yield valuable information about organ quality. Thus, activation of many developmental genes, including *Mdga2*, *Ripply3*, or tumor suppressor *Tnfrsf9*, and the oncogenic (*Tpr*, *Csnk2a1*) transcripts in the first 24 h after death [1] may increase the risk of carcinogenesis in the transplanted organs. Indeed, it is well established that recipients of transplanted solid organs have a higher risk of developing cancer than the general population [106,107]. Worrisomely, cancer accounts for approximately 10–30% of deaths in recipients who received organs [108,109]. 

Of concern is that 11 genes are upregulated both in postmortem brains and induced coma: *Cdc42*, *Csnk2a1*, *Gadd45a*, *Prdx2*, *Rasa1*, *Tnfrsf14*, *Tpr*, *Klkb1*, *Bcl2*, *Ier3*, and *Zfand2a* [108,109,110,111,112,113,114,115,116,117,118]. Further, near-death experiences and induced coma that is required in numerous conditions associated with cardiopulmonary arrest with multiple organ failure, are always accompanied by a loss of consciousness [119,120,121,122,123,124]. For neurologists, these so-called near-death experiences may have a neural basis. Thus, recent data from the brain scan of a dying human brain obtained in a non-experimental, real-life acute care clinical setting provided the first evidence that the dying brain goes through a sudden flash of memories seconds before and after the heart stopped beating and advocate that the human brain may possess the capability to generate coordinated activity during the near-death period [125].

Finally, one factor limiting the organ availability for transplantation is the religious belief. Although no religion obliges one to donate or refuse organs from deceased donors, its practice may be discouraged by Roma Gypsies, Shintoists, Native Americans, Confucians, and some Orthodox rabbis. Some South Asia Muslim ulemas (scholars) and muftis (jurists) also oppose donation of organs from deceased persons. However, they do not oppose xenotransplantation research [126,127]. More recently, organ donation for the benefit of humans in need has been seen as “posthumous giving of organs and tissues can be a manifestation of love spreading also to the other side of death” [128]. This position is assumed by the great religions of the world, especially by the Protestant, Catholic, and Orthodox Churches. Indeed, both Protestants and Catholics allow the removal of organs to save lives provided that organ removal does not violate the dignity or the eternal peace of death [129].

## Figures and Tables

**Table 1:** Upregulated genes in the brain after death and induced coma.

Gene.	Signaling Pathway, Function	Role in Body Physiology and Disease	Reference
*Gadd45a*	Key regulator of energy metabolism and longevity by regulating epigenetic DNA methylation	Key regulator of energy metabolism and longevity. Transcript levels are increased following stressful conditions	[21,22,23,24,25,26]
*Bcl2*	Blocks the apoptotic death of some cells such as lymphocytes	Stress (brain death) activates *Bcl2* for cell survival and cell death signaling pathways that converges on mitochondria. T-cell lymphomas; prostate cancer; renal cell carcinomas	[27,28,29,30]
*Ier3*	Immediate early response 3 protein involved in the protection of cells from Fas- or tumor necrosis factor type alpha-induced apoptosis	Functions either as an oncogene or a tumor suppressor in various human cancers. Downregulated in patients with cervical cancer	[31,32]
*Zfand2a*	Proteasome-mediated ubiquitin-dependent protein catabolic processes and protein targeting to ER	Involved in proteasome-mediated ubiquitin-dependent accelerated protein catabolic processes in the skeletal muscles and protein targeting to ER	[33,34,35,36,37]
*Klkb1*	Surface-dependent activation of blood coagulation, fibrinolysis, kinin generation and inflammation	Trigger complement activation. Pathophysiological hallmarks of neuroinflammatory disorders like multiple sclerosis	[38,39]
*Tnfrsf14*	Signal transduction pathways that activate inflammatory and inhibitory T-cell immune response	Inflammatory and autoimmune diseases. Tumor immunotherapy	[40,41,42]
*Csnk2a1*	Cellular processes, including cell cycle control, apoptosis, and circadian rhythm	AD pathology by regulating NR2B-mediated neurotransmission, involved in a plethora of human diseases numerous cancers, including glioblastoma	[43,44,45]
*Cdc42*	Controls cell morphology, migration, endocytosis, cell cycle progression, and epigenetic regulation	Controls diverse cellular functions including cell morphology, migration, endocytosis, cell cycle progression and epigenetic regulation. Overexpressed in tumors such as colorectal adenocarcinomas	[46,47]
*Rasa1*	Part of the GAP1 family of GTPase-activating proteins. Regulate multiple cellular signalling pathways including those that control cell growth, differentiation and survival.	Regulates multiple cellular signalling pathways including those that control cell growth, differentiation and survival Associated with basal cell carcinomas	[48,49]
*Prdx2*	Reduces hydrogen peroxide and alkyl hydroperoxides and plays an antioxidant protective role in cells.	Accelerates brain damage after stroke by activating an inflammatory response. Cancer cell proliferation	[50,51,52,53]
*Tpr*	Formation and maintenance of senescence-associated heterochromatin foci	Increased in lung cancer. Required for internal senescence-associated heterochromatin foci	[54,55]

## Data Availability

No new data were created or analyzed in this study. Data sharing is not applicable to this article.
